# Effects of Human, Relational, and Psychological Capitals on New Venture Performance

**DOI:** 10.3389/fpsyg.2019.01071

**Published:** 2019-06-18

**Authors:** Yong Wang, Cheng-Hung Tsai, David D. Lin, Oyunjargal Enkhbuyant, Juan Cai

**Affiliations:** ^1^Department of Business Administration, Huaiyin Institute of Technology, Huai’an, China; ^2^Department of Business Administration, Cheng Shiu University, Kaohsiung, Taiwan; ^3^Doctoral Program, I-Shou University, Kaohsiung, Taiwan; ^4^International MBA Program, I-Shou University, Kaohsiung, Taiwan

**Keywords:** human capital, relational capital, psychological capital, new venture, entrepreneurial performance

## Abstract

Entrepreneurship research has been focusing on intangible capitals of an entrepreneur or entrepreneurial teams. Intellectual capital has been a useful framework for researching entrepreneurship, though the inclusion of intangible capital has not been comprehensive enough. We followed and extended this stream to add psychological capital into the discussion of the relationship between intellectual capital and new venture performance. We argue that psychological capital, human capital, and relational capital are representative capitals of entrepreneurs at intra-personal, personal, and interpersonal levels, respectively, none of them can be neglected for new venture success. Based on the analyses of documentary materials of famous entrepreneurs’ interviews from trustable websites/media, this conceptual analyses with case examples found different constructs to serve as important entrepreneurial intellectual capital, which consists of human (i.e., age and education, graduate work experiences, non-graduate work experiences, role models), relational (i.e., trustworthiness and co-founder relations) capitals, and psychological capital (optimism, self-efficacy, hope, and strength). This study contributes by formalizing psychological capital as a theoretical element of intellectual capital and its effectiveness with other forms of intellectual capital on entrepreneurial performance and growth.

## Introduction

Intellectual capital is of great value to the entrepreneur as they are the basis for competitive advantages and value factors for the entrepreneurial enterprise (Nazari and Herremans, 2007). The intellectual materials mentioned include knowledge, information, intellectual asset, and experience that generate wealth (Holmen, 2005).

A gap in literature is that less has been committed in researching the role of psychological healthiness for intellectual capital in the context of entrepreneurs. The relation between the intellectual capital and entrepreneurs’ psychological wellness is an important complementation for the successful new venture performance, because new ventures do not only require entrepreneurs’ wisdom but also their psychological strengths to overcome uncertainties and difficulties (Baron, 2008). The entrepreneurial capital is denoted as the capital that the entrepreneur applies when engaging in entrepreneurial activities (Firkin, 2003), which includes both material and psychological resources (Luthans and Youssef-Morgan, 2004). As Bradley (1997) revealed organizations need to focus less on material assets and more on intangible assets. From such a viewpoint, psychological capital (Luthans and Youssef-Morgan, 2004) is also an intangible one to be incorporated and considered together with intellectual capital. For instance, Chen et al. (2018) examined that entrepreneurial behavior and decisions like those for entrepreneurial persistence need to be assessed through a knowledge and emotion lens.

In sum, the present study bridges across the research streams of intellectual capital and psychological capital, to investigate the interactive influences between entrepreneurs’ psychological and professional states. The results and discussions of the study could contribute by offering a deeper understanding of the social–psychological dynamics of entrepreneurs’ intellectual use in influencing entrepreneurial performance.

## Theoretical Foundation

Intellectual capital is a multidimensional construct that may be evaluated and researched in different contexts (Edvinsson and Malone, 1997; von Krogh et al., 1998; Youndt and Scott, 2004). In the entrepreneurship context, human and relational capitals are of most importance. Youndt and Scott (2004) described intellectual capital as the aggregate stocks and flows of useful skills and knowledge within an organization. Human capital assumes a vital part of organizations that help in increasing new information in the economy.

Some authors have researched the positive relationship that lies between human capital and its success or improved performance in the firms that deal with entrepreneurial issues (Baron and Markman, 2000; Rauch et al., 2005). Brush and Chaganti (1999) found that upcoming firms have increased performance that is related to the resources of the firm other than the approaches it employees. The intangible resource of the human capital cannot add value to the organization without back up from the managers as an important type of human capital. Pennings et al. (2017) found that human capital is highly related to firm survival and growth.

In such a sense, the psychological capital as the inner strength of human capitals should be emphasized in its role of assisting intellectual capital. The psychological resource comprises of self-adequacy, hope, strength, and positive thinking (Luthans et al., 2007) and trust (Page and Donahue, 2004). Also from such angle, psychological capital in entrepreneurial ventures can be treated as collective mental capabilities that facilitate the utilization of key knowledge.

## Methodology

Qualitative analysis is critical as many of the entrepreneurial phenomena are exploratory in nature (Gartner et al., 1994; Lechler, 2001; Gartner and Birley, 2002; Envick, 2005), when perceiving entrepreneurs as “outliers” in a business community who behave differently. Thus, the number of researched sample is not the most critical thing but what new insights could the sample tell via their explication of they own stories.

Sources of data used in this research are secondary data since researcher aims to give a comprehension of how intellectual capital (IC) and entrepreneurship can lead to the success of the entrepreneurial firm using a qualitative approach. The researcher collected and used the transcripts of total 30 entrepreneur’s interviews as secondary data for the study. All the interviews materials were collected through websites/media such as The Financial Times, Wall Street, Forbes, etc.

Five criteria were used to identify the transcripts of the entrepreneurs’ interviews to be included in the sample. First, the interviewer should be a famous or successful in the industry. Second, the source of interview must be reliable business websites and channels (Financial Times, The Economics, Wall Street, etc.). Third, the interview should have mentioned about IC and relation between success and IC area. A two-stage process to choose interviews as potential data: first one was used to identify successful and well-known entrepreneur as a quality of the participant and the other one was filtering the transcripts for those entrepreneurs have mentioned or talked about the impact of IC on their firm in the interview. In this paper it was needed to recognize successful entrepreneurs who met the sample criteria detailed above. Sources used to define entrepreneurs included media features in business magazines and broadsheet newspapers, business magazines competitions for entrepreneurs.

To benefit large-scope exploration, the selected companies represented various industries, including network, architecture, biomedical, chemicals, consumer products, consulting, education, finance, media, technology, software, investment, other services, as well as other manufacturing. The dataset is unique in providing detailed information on founders, the organizational context surrounding venture ideation as well as firm performance information. The researcher collected data through virtual documentary sources which is used of certain selected entrepreneur’s virtual interview script as main data to answer her research questions.

Moreover, to have the capacity to sum up from the sample, the researcher needed a reasonable sample size, and also the sample to mind an exceptional scope of experiences. The researcher spent 2 months working through some web-based materials via the Internet to identify potentially virtual interviews. This was quite time-consuming. The highly experienced compilers, such as the 100 most influential people in the world by Time Magazine, and the America’s richest Entrepreneurs under 40 by Forbes magazine, have been the main resource to find a potential entrepreneur. After the selection process, the researcher was able to search potential interviews as a video version and transcript version through web-based material or via Internet such as Wall Street Journal, Financial Times, Forbes Business Magazine, Entrepreneur.com, and Business Insider. Additionally, these websites/media ranked in top 15 most popular business websites as obtained from eBizMBA Rank which is continually updated average of each website’s Alexa Global Traffic Rank, and U.S. Traffic Rank from both Compete and Quantcast.

## Results and Discussion

Table 1 shows the entrepreneur’s personal information. Thematic analysis has been used to investigate the qualitative data that were obtained, following the transcriptions of the interviews. We read through the transcripts and attached codes to units of text in a manner that was as open as possible, in order to explore personal experiences and perception of an object or event.

**Table 1:** Basic information.

No.	Name	Nationality	Company	Co-founder	Net worth	Year founded	Industry	Location
1.	Reid Hoffman	American	LinkedIn	Yes	$3.1 billion	2002	VC/Internet	United States
2.	Howard Lindzon	Canadian	StockTwits	Yes	$30 million	2008	Internet	Canada
3.	Steve Forbes	American	Forbes	Yes	$430 million	1917	Media	United States
4.	Dean Kamen	American	Segway PT	No	$500 million	2001	Electric vehicle	United States
5.	Brian Halligan	American	HubSpot	Yes	$82 million	2006	Software	United States
6.	Ma Huateng	Chinese	Tencent Holdings	Yes	$24.9 billion	1998	Conglomerate	China
7.	Travis Kalanick	American	Uber	Yes	$6.3 billion	2009	Transportation	United States
8.	Diego May	Costa Rica	Junar.com	No	$240 million	2006	Software	Costa Rica
9.	Christopher Cline	American	Foresight Energy	No	$1.9 billion	2006	Mining	United States
10.	Terry Noel	American	Illinois	No	$300 million	1999	Education	United States
11.	Drew Houston	American	Dropbox Inc.	Yes	$1 billion	2007	Online backup service	United States
12.	Dave Sifry	American	Linuxcare	Yes	$500 million	1998	Technology	United States
13.	Jeff Bezos	Mexicion	Amazon.com	No	$72.8 billion	1994	Internet/retailing	Mexico
14.	Jack Dorsey	American	Twitter	Yes	$1.3 billion	2006	Internet	United States
15.	Matt Mullenweg	American	Wordpress	No	$1.5 million	2005	Software	United States
16.	Jessica Alba	American	The Honest	Yes	$1.7 billion	2011	Retail	United States
17.	Michael Bloomberg	American	Bloomberg	Yes	$47.5 billion	1981	Finance/media	United States
18.	Deena Varshavskay	Russian	Wanelo	No	$10 million	2012	Internet, E-commerce	United States
19.	Jack Ma	Chinese	Alibaba	Yes	$28.3 billion	1999	E-commerce	China
20.	Bill Gates	American	Microsoft	Yes	$86 billion	1975	Software	United States
21.	Howard Schultz	American	Starbucks	Yes	$3 billion	1971	Coffee shop	United States
22.	Larry Page	American	Google	Yes	$40.7 billion	1998	Internet/software	United States
23.	Olivia Lum	Singaporian	Hyflux	Yes	$460 million	1989	Engineering	Singapore
24.	Sara Blakely	American	Spanx	No	$1.1 billion	2000	Apparel	United States
25.	Cao Dewang	Chinese	Fuyao Glass	No	$7 billion	1987	Manufacture	China
26.	Peter Jones	British	Drangon’s Den	No	$367 million	2005	Investment	United Kingdom
27.	Chung Mong Koo	Korean	Hyundai Motor		$4.9 billion	2000	Automotive	Korea
28.	Elon Musk	American	PayPal	Yes	$13.9 billion	1998	Financial	United States
29.	Michael Dell	American	Dell Computer	No	$20.4 billion	1984	Computer hardware	United States
30.	Rob Parsons	American	Go daddy	No	$1.8 billion	1997	Domain Register	United States
31.	James Park	American	Fitbit	Yes	£6 billion	2007	Activity Tracker	United States
32.	Mark Wogan	United Kingdom	Homeslice	Yes	£4.5 million	2011	Pizza	United Kingdom
33.	Brian Chesky	American	Airbnb	Yes	$31 billion	2008	Home-sharing	United States
34.	Blaise Bellville	United Kingdom	Boiler Room	Yes	£6.5 million	2010	Live club music platform	United Kingdom

*Forbes magazine started updating net worth and ranking of more than 1,600 billionaires every trading day from 2014. The information of entrepreneur’s net worth was gathered from the list of The World’s Billionaires by Forbes magazine. 22 December 2017.*

### Human Capital

#### Age and Education

The first type of intellectual capital of entrepreneurs is human capital. Table 2 showed the information regarding the entrepreneurial human capital. In terms of education, 13 entrepreneurs graduated from university, normally setting up their company while at or after college. Two of these participants had built their first business during the college years, and eight of them dropped university before complete. Interestingly, most of them did not have any professional work experience. They were likely to have run informal ventures as teenagers. They started their business between the ages of 20 and 25 years but one of them started his first main venture at the age of 17 years. Fifteen entrepreneurs established their first business after graduating their university and gained some years of professional work experience. These entrepreneurs who had started their first venture after university had a higher level work experience in professional work with greater responsibility. They stated that having their work experience gave them more benefits than the individuals who had begun their new ventures younger. They began their companies between the ages of 27 and 38 years.

**Table 2:** Entrepreneurs information on pre-startup human capital factors.

Entrepreneur	Entrepreneurs with self-employed family	Work experience before college	University attended	Work experience after college	Age starting first venture
Reid Hoffman	No	Yes	Yes	Yes	30
Howard Lindzon	Yes	Yes	Yes	Yes	25
Steve Forbes	No	No	Yes	No	21
Dean Kamen	No	Yes	Dropped	No	25
Brian Halligan	No	No	Yes	Yes	38
Ma Huateng	No	No	Yes	Yes	27
Travis Kalanick	No	No	Dropped	No	22
Christopher Cline	Yes	No	Dropped	No	21
Terry Noel	N	No	Yes	No	22
Drew Houston	No	Yes	Yes	No	21
Dave Sifry	No	No	Yes	Yes	30
Jeff Bezos	No	No	Yes	Yes	30
Jack Dorsey	No	Yes	Dropped	No	24
Matt Mullenweg	No	No	Dropped	No	21
Jessica Alba	No	Yes	Yes	Yes	30
Michael Bloomberg	No	No	Yes	Yes	40
Deena Varshavskaya	No	No	Dropped	No	20
Jack Ma	No	Yes	Yes	Yes	31
Bill Gates	No	Yes	Dropped	No	17
Howard Schultz	No	No	Yes	Yes	33
Larry Page	No	No	Yes	No	25
Olivia Lum	No	Yes	Yes	Yes	28
Sara Blakely	No	Yes	Yes	Yes	29
Peter Jones	Yes	Yes	No	No	22
Chung Mong Koo	Yes	No	Yes	Yes	20
Elon Musk	Yes	Yes	Yes	No	24
Michael Dell	No	Yes	Dropped	No	19
Rob Parsons	No	Yes	Yes	Yes	34

Overall, the results showed that the first ventures were often the first formal businesses set up by the entrepreneurs to raise external investment. Some of the entrepreneurs ran their first informal venture as a part-time while there were at school (e.g., a school child buying jewelry to resell or a teenager designing websites for small, local businesses). These first businesses were started between the age of 15 and 26 years by entrepreneurs. Most of the studied entrepreneurs began to create human capital value to starting their first ventures some time before they really began their real business. Families were found to give significant human capital advancement to almost all entrepreneurs, however, to changing degrees and in various methods. The work and educational experiences of the entrepreneurs were quite dissimilar, both regarding quantity and quality. The two pathways are meant to be seen the common design of pre-start-up human capital associated with certain types of pre-start-up experience and with starting a business at a specific age.

Some of entrepreneurs credited growing up with entrepreneurial relatives as a motivation for turning into a business person, no member said that school had given them motivation to seek a career as entrepreneur. No entrepreneur who had a business degree, even where they figured this may have been to school degree helpful, said that in the event that they had not done their degree they couldn’t have begun their business. Twenty-nine out of 30 entrepreneurs went to university, while 8 of them dropped out from university. Those who had graduated from business school or a computer science program could give technical knowledge and professional value to starting up their business.

“*We were afraid if we waited, someone else would beat us to it. It was a hard decision and I know my parents had their concerns. And while I would never encourage anyone to drop out of school, for me, it turned out to be the right choice*.” (Gates)

#### Inspiration and Role Models

The finding shows that a parent role model can be a direct influence to the success of the children (see also Marshall, 1998). Entrepreneurial parents and other family members are ascribed with motivating career choices, which could be termed formally as observational learning through role modeling (Shapero and Sokol, 1982; Bowen and Hisrich, 1986; Mokry, 1988; Scherer et al., 1989).

“*My dad, who is also entrepreneur, would give me money to buy the jewelry. I started selling Native-American jewelry that became popular that time. I went to Mexico, to buy the stuff then I’d sell the jewelry up in Toronto. It was like a license to print money. Also I would set up shop in my house and sell to all his friends and their kid. I had some advantage other kids didn’t have*.” (Howard)

The findings showed that the entrepreneur’s parent role model influences their career hopes and education aspirations.

##### Non-graduate work experience

Human capital is the experience and commitment of staff, as well as their skills that are used in the organizations (Penrose and Penrose, 1995). Human capital is crucial in any organization, but it is low in value if the managers and other entrepreneurs using it do not use it as it is supposed to be used (Marr and Roos, 2005; Oviatt and McDougall, 2005). Although it is considered as a lesser factor in the organization, the importance of human capital in an organization found that it is the most important resource regardless of being intangible (Edvinsson and Malone, 1997; Sveiby and Simons, 2002; Becker and Huselid, 2006).

The data suggest that almost all entrepreneurs had some kind of experience working for someone else at a young age.

“*I think it’s fair to say I had absolutely no idea what I would do. I went in thinking I wanted to study physics, but German was a requirement, so I changed to the engineering school. The whole idea of college is to expose yourself to various ideas and cultures and places, to minor in something different, to travel abroad. The part that’s most important in an education is how to deal with people. There’s no job I know that you do by yourself, and I learned as much from the two guys I worked for at Salomon Brothers, Billy Salomon and John Gutfreund, as I’d learned at Harvard. In the end, it’s people skills that you need*.” (Bloomberg)

The finding shows that those entrepreneurs who graduated from university normally continued with a part-time job during the university years. All the entrepreneurs had different work experiences such as a small retail shops, Chinese restaurant, modeling, doing a paper round, and also working in someone’s home. The general human capital they created included increasing different workplace experience, creating client benefit aptitudes, working with partners, and, in Howard’s case, developing customer service skills.

Two of the entrepreneurs started their first venture before age 20 years which is considered as an informal venture. However, half of the total entrepreneurs which is 14 out of 30 had taken part in informal ventures when they were teenagers before building their own first ventures. These below cases show that entrepreneurs saw informal ventures could be a way of earning money and becoming familiar with venturing processes.

“*Learning to program at around the age of 12 made me feel intellectually powerful and creative in profound ways. I have been teaching children to program since I was a teenager myself*.”(Jack)

“*At age 12, I taught myself computer programming and created a video game called Blastar, which I sold for $500*.” (Elon)

Informal venturing was normally appraised as being more important in developing entrepreneurial skills than faculty, and was often times considered being a crucial place to begin for an entrepreneur’s career like alternative young entrepreneurs who started their informal ventures on the idea of a private interest. These informal ventures give a chance to observe enterprise trial skills at comparatively little chance of financial risk to the young business founder.

##### Graduate work experience

The contribution of human capital in an organization makes the issue of recognizing the extent of the resource that should be incorporated and the one that ought not (Andriessen, 2004). In this way, the rule implies that while identifying the knowledge of human capital there should be an appropriate assessment of resource encounters and abilities obtained that can be helpful to the firm (Stuart, 1990; Bosma et al., 2004; Rauch et al., 2005). Studies have consistently noted that previous entrepreneurial experience has an important impact on business performance of an entrepreneur’s current venture (Gimeno et al., 1997; Ucbasaran et al., 2006).

In the information gathered in this study, most of the entrepreneurs undertook graduate level work after university and 15 of the remaining entrepreneurs who graduated from university had either more usually started his or her first business after school or found their business during the school years. Entrepreneurs who took part in professional work did various type of jobs such as sales, recruitment, the technology industry, management work, restaurant, and consulting, and they were frequently ready to utilize their work experience after graduation to clarify the positive and negative lessons it had shown entrepreneurs in needing to seek after an entrepreneurial career. Entrepreneurs with post-graduate work experience were less likely to have undertaken entrepreneurial ventures than those entrepreneurs who established their younger businesses during or shortly after university school, including those who had faced some negative lessons from their graduate professional work.

“*At Salomon, I was ‘demoted’ as head of equity trading and sales to head the emerging computer systems area. If I hadn’t gotten fired from Salomon, which became part of Citigroup, I wouldn’t have gotten a $10 million severance, used my electrical engineering degree to begin my own information technology company and program a computer terminal for bond traders*.” (Bloomberg)

Anyhow, with post-graduate work experiences, entrepreneurs learned to transfer important skills, solve problems, and answer questions.

### Relational Capital

#### Trustworthiness

Strong relationships are often grounded on trust between cooperative persons (Coleman, 1990; Larson and Starr, 1993). Thus, the relational dimension of intellectual capital concerns interpersonal relationships developed through a series of interactions (Granovetter, 1992). It gives the focus on the special relationships that people have, such as trust, respect, and kindness.

Among these factors, trust is the forerunner to the acquisition of resources, exchange, and the combination of knowledge. Therefore, it is likely that someone who develops a higher level of trust can appropriate the knowledge, information, and other forms of resources accessible in his relational network. In other words, relational capital will substantially increase someone’s propensity to the business.

“*A more important problem is the trust factor. The investors were only concerned with chasing returns and not investing with a great manager who really knew what he was doing – someone you could be with for 10–15 years and it didn’t matter what he did today or six months from now because you were going to invest in this guy for 10 years just like you would a business*.” (Jeff)

As expressed, the most major resource alludes to the support received from the informal networks of the entrepreneur, including their family, friends, and as well as their life partner. Networks are made between the customer and company, in addition to its supply agents and other vital parts of the business system. This has increased additional participation between the company and its providers with clients. Entrepreneurial goals will be the most grounded, and the prospect of entrepreneurial actions will be the most elevated, when there is a high level of self-efficacy coming from both the nearness of solid social influences from personal connections.

#### Co-founder Relations

A main choice for entrepreneurs was whether to create a new venture alone or find a co-founder as a business partner. Sixteen entrepreneurs analyzed in this study had co-founders who built their businesses together during the entire journey of their first big business. In each of these cases, the two co-founders assumed executive functions in the operation of the company, instead of being merely a non-executive director. Entrepreneurs met with their business partners through different media (e.g., a pre-existing friendship, relatives, and business networking). Having a strong and trusting relationship with a business partner was considered very important by most entrepreneurs who were fellow co-founders of a new venture. Having a co-founder was proven as a helpful source of support for the entrepreneur’s solitary journey.

“*I think the most important thing is having a support network to be able to speak to, and people who can help you through tough times. I’m very lucky to have a business partner, another great thing to have. You’re not there alone in hard times, someone to give you a pat on the back*.” (Sara)

Moreover, the work done by the relatives and friends amid the main years of business helping entrepreneurs to make up for the money-related limitations and also lessen the cost used to pay staffs during the starting period (Sanders and Nee, 1996). The help by family and friends offer security to business persons, which benefits the exercises embraced by the new firm. With this respect, the image of the young venture in the market is an essential piece of the intangible asset that can affect the organizations in numerous measurements. Consequently, a positive image of a firm is essential as it empowers the firms to draw in clients and also keep up their loyalty in the beginning times of building up a firm (Shane and Cable, 2002), especially those built through a good co-founder relationship.

“*Me and my brother, we started the business together, so I guess he has a very similar entrepreneurial spirit and interest in the same things as me. So I guess we wouldn’t have done the business if there hadn’t been the both of us. He was more on the technical side, and I was more on the kind of entrepreneurial and business side. I don’t think we would have done it on our own without the other. He would have probably been happy just messing around and programming and stuff. I was really interested in getting into the business side*.” (Elon)

The importance of the relationships between business co-founders was noteworthy, as it offered help, support, trust, and a person to talk about the company who was understanding and engaged. As far as human capital, entrepreneurs were specific about the human capital that they and their partner brought to the company, and each co-founder came up with different set of skills and knowledge. On the contrary, those who had started their business solo were more likely to remember that he was an entrepreneur as a solitary career compared to those who had a business partner.

### Psychological Capital

It is a component that is made up of four important aspects, namely self-adequacy (Bandura, 1997), hope, strength, and positive thinking (Carver and Scheier, 2003). Psychological capital means that other important factors are paramount to the success of an organization other that financial and material assets. Some of the research on the components of psychological capital such as positive thinking and self-efficacy plays an important role in the entrepreneurial behavior and success (Gartner, 2007; Hmieleski and Baron, 2009).

In alignment with our initial prognosis, it has been discovered that the psychological capital of entrepreneurs shares a positive relationship with the performance of their new ventures. Maybe the most encouraging thing about the findings of this study is that the psychological capital can most likely be the development of individuals, which highlights the opportunity to train entrepreneurs so that they can flourish and even flourish in difficult situations. Many people may develop professional skills and behaviors, but some people have particular characteristics that improve their potential as entrepreneurs.

#### Optimism

Kirby (2006) states that the investigation of the psychological literature of entrepreneurs, arguing that, “The main psychological characteristics of the entrepreneur would appear to be risk-taking ability, need for achievement, locus of control, desire for autonomy, deviancy, creativity and opportunism, and intuition.” To a certain degree, this all relies on optimism. More and more publications and literature suggest that entrepreneurs’ emotions can have a significant influence on their ability to grow and develop their new businesses. “Considering the great emotional demands that are placed on most entrepreneurs” (Baron, 2008).

“*Well, first of all I’d say I actually think I feel fear quite strongly. So it’s not as though I just have the absence of fear. I feel it quite strongly. There are just times when something is important enough that you believe in it enough that you do it in spite of fear*.”(Elon Musk)

It is characterized as having the positive anticipation of a result (Carver and Scheier, 2003). In any case, self-viability is an individual trait that is created through life encounter (Bandura, 1977). Hopefulness has appeared to be available in people who have connected with people who have both experienced time and setting of life (Schulman et al., 1993). Individuals who are viewed as hopeful people do not surrender easily but adapt to present circumstances exhibited to them in this way staying occupied with the quest for their objectives not considering the severity of the challenge while their pessimist counterparts have worries in case of a challenge and they can easily despair.

“*In my soul I believe that optimism is rewarded. I truly believe in karma. I hang around with positive people who don’t keep score – which limits the people you hang around*.” (Howard)

“*Hyflux has an energetic culture that c rooted in entrepreneurship. We have the boldness to dream. We have a ‘can-do’ spirit. Obstacle can be overcome; it’s how we approach the challenges and solve the problems*.” (Olivia)

#### Self-Efficacy

This study suggests that the self-efficacy of individual firms, described as a person’s belief in his ability to achieve a task, affect the development of business action, behavior, and intentions. The presence of an entrepreneur role model positively affects the degree of business self-efficacy. Self-efficacy is a valuable built in characteristic clarifying the dynamic procedure of assessment and decision that encompasses the improvement of entrepreneurial hopes and the consequent choice to occupy in entrepreneurial behavior. What’s more, people may frame business hope earlier in their careers, yet not follow on those aims until the point when the enactive area procedure gives the level of certainty expected to accomplishment in a new venture.

“*When I got into high school, I got social again, like really wanted to be able to go out and engage with the people. And I realized that wow, there are all these skills, these things that I can help people, they would come to me and they was like, ‘We have these consulting projects like could you build this for me’? And I was like, wow, this is fabulous, this is more money than my parents have ever given me. And so I got really bitten by the entrepreneurial bug.*” (Dave)

People can give the largest amount of performance in any tasks they embrace in their lives (Bandura, 1977). It is a characteristic that is considered as the state as it increases with increase in experience of a specific activity. People found to have a positive state of self-adequacy consistently set objectives that go for testing issues in their lives and working toward accomplishing the objective in unpleasant and troublesome circumstances (Phillips and Gully, 1997). Baum et al. (2001), Baum and Locke (2004), and Hmieleski and Corbett (2008) have discovered a positive association between self-adequacy and the improvement of firms. Anna et al. (2000) found that there is a close connection between self-adequacy and work-related interests and choice of occupation among the students in institutions of higher learning despite their sexual orientation. Along these lines, the self-adequacy is a fundamental part of a business enterprise that can be framed at the underlying phases of the business person as a profession. Additionally, it is sufficient in picking the purposing of the firm when thinking of one.

An interesting finding from this thesis is that people who observe a low-performing parenting model also have hopes of pursuing a professional career, even though their self-efficacy and aspirations for education and training may be lower than those high performance parenting model an entrepreneur who has had success at work can experience higher levels of self-efficacy in similar circumstances in new situations. This person can set higher personal goals, be more persevering to overcome obstacles, and have better long-term performance.

“*I think the one I would put at the very top of the list is independent mind. I think that, in order for someone to succeed as an entrepreneur, he or she has to be willing to trust that his or her convictions are sound. It means having the confidence to think that I am in the minority on this idea, and I am okay with it*.” (Dave)

The findings showed that the psychological capital of entrepreneurs is positively linked to the new venture’s success. From the observation that psychological capital can provide entrepreneurs with the psychological resources to face the emotional challenges intrinsic in the entrepreneurial action. The highest are the degree of business self-efficacy in the early stages of the business the greater are the strong business intentions. The more prominent the level of entrepreneurial self-efficacy originating from social influence, the more grounded the entrepreneurial intentions, and the higher the likelihood prospect of entrepreneurial activities. The higher the level of entrepreneurial self-efficacy and the higher the level of objective defining and goal responsibility, the more grounded the entrepreneurial goals.

#### Hope

Snyder (2000) and Hmieleski and Corbett (2008) characterize trust in the enterprise as the part of a person to battle against any troubling issue while participating in activities of an organization. Business persons who are high in hope can handle and adapt to situations that are sudden or have a shocking misfortune (Lopez et al., 2003). It is a method of dealing with stress particularly for individuals faced with distressing conditions.

“*People management is important – and this encompasses motivation, development, rewards, and retention – as it is the people in the company who can help you succeed*.” (Peter)

The hope is considered to have a level of significance in the entrepreneurial setting as it helps in defining objectives and making arrangements for future, which helps with accomplishing the goals of a firm (Alexander and Onwuegbuzie, 2007).

“*I want my investors to say I busted my ass to get them their money back plus something*.” (Howard)

From time to time, hope may also come from the desire toward a leader or mentor to offer constructive guidance.

“*We will need to put in a lot of effort communicating with mid-level management and developing a system of mentoring the younger staff. With time, the communication avenues, common processes, and management systems that are being developed will be integrated in transmitting our vision, mission, and values to our entire staff across the different markets*.” (Olivia)

#### Resilience

Resiliency and flexibility are the capacity of people to change distressing life encounters and adjust to the changing unpleasant life requests (Tugade and Fredrickson, 2004). Individuals who are flexible can survive any conditions as they learn ways of adjusting to various encounters of life. Masten et al. (1999) states that to term individuals as strong, they are likely to have encountered life threatening experiences, which they overcame by going through the situation.

“*I think probably the biggest factor that separate successful entrepreneurs from those who die on the vine is that they just decide they will do no matter what. They may have to change their approach to an idea, but they have to be resilient enough to get up, dust themselves off and go at it again*.” (Howard)

The capacity of these people to conquer the threatening circumstance using different approaches is a characteristic that strong people have (Markman et al., 2005). Business people are faced with unpleasant occasions in their exercises and to conquer these conditions they ought to have the capability of bouncing again from the assorted variety and utilize the accessible assets to make sure they succeed.

### The Mutual Influences Among Psychological, Human, and Relational Capitals

One of the most contributive parts of this paper is the finding that the three major capitals in the entrepreneurship context may have mutual influences on one another. Although we only retrieved some initial evidences from the sampled interview contents, the theoretical and practical implications brought by the interactions among human, relational, and psychological capitals are important. We offer some discussions of the important interactions as follows.

The first important mutual influences we found in the data are the influence of self-efficacy (psychological capital) on education (human capital). Self-efficacy, as in the provided example below and other famous entrepreneurs’ success stories, is often a major factor that affects the nurturing of prospect entrepreneurs’ capability. However, the direction of influences is not always “positive.” In some cases, self-efficacy did not lead the entrepreneur to be educated further; on the contrary, self-efficacy might encourage entrepreneurs to drop from their current education.

“*… Trust your gut … I dropped out just before finals because I got an internship at Morgan Stanley. As an entrepreneur, you have to take risks. But as an engineer, there’s always a backup*.” (James P.)

Psychological capital is also influential on relational capital of entrepreneurs. An example in our analyses is that hope once helped an entrepreneur to build relationship with their new customers.

“*The best part of a pitch is passion … We did a very poor pitch to our first landlord: a four-page PowerPoint presentation and some pizza. An experienced restaurateur would have said, ‘They don’t have a clue.’ But we were passionate. Starting out, to win a pitch you need to get your passion across*.” (M. Worgan)

It is interesting that we found both resilience and self-efficacy are influential on co-founder relations, positive and/or negative. In a quote from the founder of Airbnb, the entrepreneur expressed that resilience as a strong psychological attribute and co-founder relations are of mutual effects, while in other quote from the Pinterest’s founder saying that self-efficacy also as a strong psychological factor might have relatively less influence on co-founder relations by putting too much self-assertive thoughts on the overall directions when making collective decisions.

“*The turning point for Airbnb was when my two cofounders and I lived and worked together seven days a week. Bouncing ideas around late at night formed some magical moments and that close bond is what builds your company*.” (B. Chesky)

“*Don’t take too much advice. Most people who have a lot of advice to give – with a few exceptions – generalize whatever they did. Don’t over-analyze everything. I myself have been guilty of over-thinking problems. Just build things and find out if they work*.” (B. Silbermann)

It is interesting that we found that keeping optimism as a kind of psychological capital might overcome some potential flaw brought by the feelings of uncertainties that are due to insufficiencies in human or relational capital aspects.

“*One misconception is that entrepreneurs love risk. Actually, we all want things to go as we expect. What you need is a blind optimism and a tolerance for uncertainty.*” (D. Houston)

“*Put the vision first … When you first go into something creative, it’s a mistake to focus on the money. Do it for the right reasons and it’s more likely to turn into a business than if you try and force it*.” (B. Bellville)

Also encouraging was that being a good state of psychological capital could help overcome negative relations. As an entrepreneur noted strongly,

“*Don’t be afraid to assert yourself, have confidence in your abilities and don’t let the bastards get you down.*” – Michael Bloomberg, Bloomberg L.P. founder

Additionally, we’ve found supports indicating that relational capitals could facilitate psychological capital. Such relational support may come from horizontal or vertical relations.

“*Get a mentor in the applicable field if you’re at all unsure of what you’re looking for.*” – Kyle Bragger, Forrest founder

“*No matter how brilliant your mind or strategy, if you’re playing a solo game, you’ll always lose out to a team.*” – Reid Hoffman, LinkedIn co-founder

In sum, some exploratory but promising findings could encourage us to think and examine more on the mutual influences and co-evolutions of human, relational, and psychological capital.

## Conclusion

In conclusion, for entrepreneurs to have psychological capital, they should possess the four main components that comprise hope, resilience, optimism, and self-efficacy. Entrepreneurs with psychological capital tend to have a reputation for mental hardiness that attracts customers as well as situations that enable the organization to reinforce their capacity within the organization. Entrepreneurs with these characteristics do not only increase the well-being of their organizations or businesses but also allow the organization to develop their grit with the aim of preserving entrepreneurial process (Hmieleski and Ensley, 2007). These psychological capital components target the emotional demands of the organizational behavior and success, therefore, acting as an essential resource that should always be available to all entrepreneurs that start new ventures.

The objective of this study has been to illustrate the relationship between the success of new firm and intellectual capital of entrepreneur. This part of the conclusion analyses the findings in connection to the current literature, to give answer to the thesis questions. In addition, this research examined the complex relationships between particular factors of IC, structural capital, human capital, and relational capital, in link to the firm’s success and the entrepreneur. The main finding was that the development of these sort of IC factors and even though the main beneficiary of existing relational capital and human capital, and psychological capital and the entrepreneur is on one side the major factor responsible for the creating new venture.

The findings show that the IC of new venture is positively connected with the success recognized by the entrepreneur. The partial findings uncover that both the relational capital and human assume an essential part in the growth of new business while the structural capital is generally important but this study couldn’t find relatively enough evidence for structural capital from the interviews. This was the main weakness of this study. As mentioned above, this research provides the dimensions of IC (relational capital and human capital) and PC that are main factors for entrepreneurial firm success.

Human capital is the entrepreneur’s essential quality that alludes to her or his business skills, abilities, and knowledge that come from training, experience, and education. Generally, human capital is the learning knowledge, abilities, and traits created by a person which can be utilized as both an entrepreneur and a worker, and can be produced in any segment of the economy. It can be learned through instruction, work, and education. Entrepreneurship, especially human capital, are the capacities, learning new abilities, collecting knowledge, and quality created by people that are particularly essential to be an entrepreneur, particularly the experience of being a businessman. The main focus of this research involved the family has been the impact of the parents’ profession on their children’s decision to be an entrepreneur.

The findings suggest that entrepreneur’s emotions can have an important influence on their capacity to build and develop their new business. In addition, the advantages of psychological capital noticeable to be as significant to entrepreneurial success as more traditional dimensions of psychological capital. This perception might be due in part to the fact that entrepreneurs generally face scarcity of human capital, social capital, and also financial capital. Psychological capital can be a censorious form deciding why some businessmen, but not others, can endure and create successful startups with limited resources.

## Author Contributions

YW conceptualized the manuscript and is in charge of R&R works. OE developed the first draft. C-HT and DL conducted the qualitative analyses. JC edited and reviewed the manuscript.

## Conflict of Interest Statement

The authors declare that the research was conducted in the absence of any commercial or financial relationships that could be construed as a potential conflict of interest. The handling Editor declared a shared affiliation, though no other collaboration, with one of the authors C-HT.
